# The Effect of Business Operating Systems on Nursing Home Termination

**DOI:** 10.3389/ijph.2023.1605439

**Published:** 2023-02-01

**Authors:** Xueying Jin, Kazuaki Uda, Miho Ishimaru, Tomomi Kihara, Takehiro Sugiyama, Kazumasa Yamagishi, Hiroyasu Iso, Nanako Tamiya

**Affiliations:** ^1^ Department of Social Science, National Center for Geriatrics and Gerontology, Obu, Japan; ^2^ Health Services Research and Development Center, University of Tsukuba, Tsukuba, Japan; ^3^ Department of Health Services Research, Institute of Medicine, University of Tsukuba, Tsukuba, Japan; ^4^ Department of Oral Health Promotion, Graduate School of Medical and Dental Sciences, Tokyo Medical and Dental University, Tokyo, Japan; ^5^ Department of Public Health Medicine, Institute of Medicine, University of Tsukuba, Tsukuba, Japan; ^6^ Institute for Global Health Policy, Bureau of International Health Cooperation, National Center for Global Health and Medicine, Tokyo, Japan; ^7^ Diabetes and Metabolism Information Center, Research Institute, National Center for Global Health and Medicine, Tokyo, Japan

**Keywords:** nursing home termination, long-term care insurance, financial incentives, additional payment, business operating system

## Abstract

**Objectives:** Nursing home terminations have increased worldwide due to rising costs, staffing shortages, and the coronavirus disease pandemic. However, little is known about the impact that business operating systems have on nursing home termination.

**Methods:** This study used the National Long-term Care database, which comprised 7,842 operating nursing homes in January 2018. Nursing home termination was identified when nursing homes discontinued provision of long-term care services to all residents between January 2018 and December 2020. Business operating systems that were reimbursed by the LTC insurance system were the exposure of interest. The logistic regression model for nursing home termination included a series of organizational, internal, and external factors as covariates.

**Results:** From 2018 to 2020, 83 (1.1%) nursing homes were terminated. The proportion of reimbursed nursing homes varied greatly depending on the type of business operating systems. Implementing physical function training and improving working conditions were significantly associated with a lower risk of nursing home termination.

**Conclusion:** Financial incentives to several business operating systems are an effective way to build a sustainable environment for nursing homes to continue to exist.

## Introduction

Nursing home terminations negatively affect residents’ cognitive and functional status because they no longer receive consistent and reliable services (1). Faced with growing pressure from rising costs, staffing shortages, and the coronavirus disease (COVID-19) pandemic (2), nursing homes are shutting their doors at a rapid pace (3). The availability of nursing homes is insufficient, especially in countries like Japan where the average number of people on a waiting list to enter a nursing home was 100 people in 2019 (4). Nursing home termination may lead to severe consequences for the whole care market. Long-term care (LTC) provider terminations have increased annually, with 2020 being the most significant year to date (5).

There are many causes for nursing home termination. These causes can be grouped into organizational, internal, and external factors (6–10). Organizational factors are the structural characteristics of the nursing home, including size, number of years in business, and ownership (11). Internal factors are labeled as operating characteristics, including business operating systems, staff mix, and resident case-mix. External factors are factors such as areas with high income, high market competition (8, 12), and the COVID-19 pandemic. Most prior studies have examined organizational and external factors (6, 9, 13); however, only a few have considered internal factors (8). Notably, past studies have proposed the importance of operating characteristics’ influence on nursing home termination (7, 14); unfortunately, almost none have explored this influence (7–9, 11) because of difficulties in ascertaining the relevant information.

“Business operating systems” refers to a collection of tools and processes that serve as standards throughout an organization: their goal is to maintain organizational consistency and increase efficiency. A successful business operating system has clear objectives, shared values, requirements, and actions to achieve goals (15, 16). Japan’s LTC insurance system emphasizes the value obtained from operational strategies linked with higher quality care (10). The government has introduced several business operating systems and makes additional payments when nursing homes are equipped with these business operating systems. Although financial incentives were first implemented 20 years ago, little is known about the impact of these business operating systems on nursing home termination.

Owing to the universal coverage of LTC, this study utilized the national database of LTC insurance claims, which contains detailed information on both resident and nursing home characteristics. In Japan, there are three types of LTC facilities: special nursing homes (i.e., a living facility for those who do not have severe medical conditions), geriatric intermediate facilities (i.e., providing professional rehabilitation to enable residents to return home), and LTC medical facilities (i.e., medical-based facilities for those who need long-term medical care) (10). Our study only focused on the termination of special nursing homes because the other two types of facilities are transitioning into a new type of facility following a new policy in 2018. This study aimed to investigate the impact of business operating systems on nursing home termination by controlling for both resident and nursing home characteristics.

## Methods

### Data Source and Participants

Our study utilized the national database of LTC insurance claims from the Ministry of Health, Labour and Welfare under the guideline of third-party provision. All nursing home registration records were included in this database, which contained information on nursing homes’ date of inception, number of residents, and corporation type. In addition, it recorded the residents’ demographic characteristics, including age, sex, level of care required, and services received. Notably, the database stores all the reimbursement records for the business operating systems. In January 2018, 7,842 nursing homes were in operation. This study was approved by the ethics committee (approval number: 1698).

#### Outcome

We defined nursing home termination as nursing homes discontinuing their provision of LTC services to all their residents for any reason (i.e., change of ownership or closure) from 2018 to 2020. To ensure the accuracy of the outcome, we confirmed whether these nursing homes had been removed from both the LTC claims and nursing home registration databases.

#### Organizational Factors

The number of residents at baseline was used as a proxy for nursing home size. The type of corporation (social welfare corporation vs. government-owned vs. others), number of years in business, and types of care (traditional vs. unit) were included as the organizational factors. Traditional care is mainly provided in nursing homes in a shared-room setting. In contrast, unit care refers to person-centered care for a small number of residents (<10) living as one unit, where care is mainly provided in private rooms (17).

#### Internal Factors

##### Business Operating Systems

The nine business operating systems which the LTC insurance system reimbursed with additional payments were explored, and the relative requirements (18) of these additional payments are described in Table 1. Business operating systems are presented as binary variables: receiving reimbursement or not receiving reimbursement at baseline.

**TABLE 1:** Reimbursed business operating systems and requirements, Japan, 2018–2020.

Business operating systems	Requirements determined by the Ministry of Health, Labour and Welfare	Additional payments
Regular medical guidance by a psychiatrist	Half of the residents have dementia, and a psychiatrist provides regular medical guidance for dementia at least twice a month	25 units per day
Nutritional management	There is a full-time registered dietitian to create and manage nutrition plans for residents individually	14 units per day
Strengthening of the services provision system	The proportion of certified care workers among care workers is more than 50%	18 units per day
Palliative care system	All the following requirements must be met: i) 24-h contact in cooperation with nursing staff	160 units per day
	ii) Discuss and review end-of-life care plan	820 units from 2 days before death
	iii) Provide staff training on end-of-life care	1,650 units on the date of death
	iv) Care provided in private rooms	
Individual functional training system	At least one full-time functional training instructor prepares individual functional training plans and systematically implements functional training	12 units per day
Young-onset dementia care system	Admit residents with young-onset dementia and provide specialized services for them	120 units per day
Sufficient night shift staff	The number of caregivers and nurses exceeds the night shift assignment standard	18–27 units per day
Therapeutic diet	Therapeutic diets are formulated by doctors and provided under dietitians’ instruction	18 units per day
Improvements in working conditions	Implement a wage increase system based on care workers’ experience and qualifications	37,000 Japanese yen per caregiver per month

Source: Abe T. Perfect reimbursement guides for long-term care (2015) 2015. Igakutushinsya.

Note: One unit equals 10 Japanese yen.

##### Resident Case-Mix

The characteristics of nursing home residents influence nursing homes’ necessary staffing, making them relevant to ongoing operations (7). For resident case-mix, we included the proportions of residents aged ≥85 years, female residents, and residents needing severe level care as covariates.

#### External Factors

The Herfindahl-Hirschmann Index for 2018 was included to show market competition, which was calculated by summing the square of market share for each nursing home in a prefecture. We used an extra-regional charge rate as a proxy of regional income level for the amount of LTC services utilized. The government has determined eight regional levels for extra charges according to the labor costs of local government employees; the extra rates in specific regions are as follows: level 1 (20%), level 2 (16%), level 3 (15%), level 4 (12%), level 5 (10%), level 6 (6%), level 7 (3%), and level 8 (0%). Three regional labor cost levels, categorized as high (levels 1–3), middle (levels 4 and 5), and low (levels 6–8), were included in our analysis.

### Statistical Analysis

Chi-square or t-tests were performed to compare the organizational, internal, and external factors between closed and still operational nursing homes. A logistic regression analysis was performed to explore the determinants of nursing home termination. Highly correlated variables (>0.7) were excluded to avoid multicollinearity. Data management and analysis were performed using STATA version 16, and *p*-values <0.05 were regarded as statistically significant.

## Results

Between 2018 and 2020, 83 nursing homes terminated their services, accounting for 1.1% of the 7,842 nursing homes that were operational in January 2018. Table 2 summarizes the statistics for independent variables by terminated and operating nursing homes. On average, 67.7 residents resided in nursing homes, and 95.4% of nursing homes were social welfare corporations, with a mean of 16.2 years in business. The proportion of reimbursed nursing homes varied greatly depending on the types of business operating systems. The most frequently reimbursed operation systems were improvements in working conditions (91.1%), nutritional management (89.1%), and sufficient night shift staff (84.4%). In contrast, only a quarter (24.1%) were reimbursed for strengthening the service’s provision system.

**TABLE 2:** Description of nursing home organizational, internal, and external factors at baseline, Japan, 2018.

	Total	Continued operation	Termination	*p*-value
	*n* (%)	*n* (%)	*n* (%)	
	7,842 (100)	7,761 (98.9)	83 (1.1)	
Organizational factors				
Number of residents (mean, SD)	67.7 (26.9)	67.9 (26.9)	56.2 (24.5)	<0.001
Types of corporation				
Social welfare corporation	7,483 (95.4)	7,424 (95.7)	59 (71.1)	<0.001
Government-owned	279 (3.6)	256 (3.3)	23 (27.7)	
Number of years in business (mean, SD)	16.2 (5.8)	16.2 (5.8)	16.8 (5.5)	0.337
Type of care				
Traditional	4,584 (62.0)	4,797 (61.8)	61 (73.5)	0.029
Unit	2,984 (38.5)	2,962 (38.2)	22 (26.5)	
Internal factors				
Resident case-mix (mean, SD)				
Proportion of residents ≥85 years old	30.1 (9.6)	30.6 (10.6)	34.9 (12.7)	0.022
Proportion of female residents	79.5 (6.7)	79.4 (6.7)	79.7 (7.0)	0.810
Proportion of severe care need level	30.6 (10.6)	30.1 (9.6)	32.4 (9.3)	<0.001
Business operating systems				
Regular medical guidance by psychiatrists	2,223 (28.4)	2,198 (28.3)	25 (30.1)	0.720
Nutritional management	6,984 (89.1)	6,917 (89.1)	67 (80.7)	0.014
Strengthening of the services provision system	1,893 (24.1)	1,873 (24.1)	20 (24.1)	0.990
Palliative care system	5,741 (73.2)	5,690 (73.3)	51 (61.4)	0.015
Individual functional training system	4,448 (56.7)	4,419 (57.0)	29 (34.9)	<0.001
Young-onset dementia care system	3,466 (44.2)	3,435 (44.3)	31 (37.3)	0.210
Sufficient night shift staff	6,619 (84.4)	6,559 (84.5)	60 (72.3)	0.002
Therapeutic diet	6,208 (79.2)	6,151 (79.3)	57 (68.7)	0.018
Improvements in working conditions	7,145 (91.1)	7,085 (91.3)	60 (72.3)	<0.001
External factors				
Herfindahl-Hirschman Index	67.7 (44.6)	67.7 (44.5)	74.7 (48.9)	0.150
Regional labor cost level				
High	1,268 (16.2)	1,257 (16.2)	11 (13.3)	0.300
Middle	4,646 (59.2)	4,588 (59.1)	56 (67.5)	
Low	1,930 (24.6)	1,914 (24.7)	16 (19.3)	

Note: The *p*-values were calculated using a Chi-square test for categorical variables, t-test for continuous variables.

SD, standard deviation.

With regard to organizational factors, nursing home facilities that continued to operate were larger than those terminated: the average number of residents in continuously operated nursing homes was 67.9, compared to 56.2 in nursing homes that were terminated. Compared to nursing homes that continued to operate, a significantly higher percentage of terminated nursing homes were government-owned (27.7% vs. 3.3%) and provided traditional care (73.5% vs. 61.8%). Terminated nursing homes had more residents over the age of 85 and more people with severe care-need levels than facilities that continued to operate. Government-determined business operating systems were implemented more frequently by continuously operated nursing homes than by terminated nursing homes; these systems included nutritional management, palliative care, individual functional training, sufficient night shift staff, therapeutic diets, and improvements in working conditions.

Table 3 presents the logistic regression results for factors affecting nursing home termination. After controlling for organizational, internal, and external factors, two business operating systems were found to reduce the risk of nursing home termination: individual functional training systems (odds ratio [OR] 0.54, 95% confidence interval [CI; 0.32–0.85]) and improvements in working conditions (OR 0.54, 95% CI [0.30–0.96]).

**TABLE 3:** Business operating systems associated with nursing home termination: logistic regression results, Japan, 2018–2020.

	Odds ratio	95% confidence interval	*p*-value
Business operating systems			
Regular medical guidance by a psychiatrist	1.65	(0.97–2.79)	0.064
Nutritional management	0.92	(0.49–1.7)	0.779
Strengthening of the services provision system	0.86	(0.50–1.51)	0.607
Palliative care system	0.80	(0.49–1.31)	0.376
Individual functional training system	0.52*	(0.32–0.85)	0.010
Young-onset dementia care system	0.97	(0.60–1.56)	0.887
Sufficient night shift staff	0.87	(0.50–1.52)	0.630
Therapeutic diet	0.83	(0.49–1.4)	0.484
Improvements in working conditions	0.54*	(0.30–0.96)	0.037

Note: The model additionally adjusted for organizational factors (number of residents, types of corporation, number of years in business), internal factors (proportion of residents ≥85 years old, proportion of female residents, proportion of residents with severe care level), and external factors (Herfindahl-Hirschman Index, labor cost level).

Statistical significance: *p < 0.05.

## Discussion

Given the global aging population, making the LTC market sustainable is an international concern. In Japan, 83 nursing homes (1.1%) were terminated over the study period, that is, from 2018 to 2020. Our study provides insights into nursing home business operating systems and examines their effects on nursing home termination. Implementing individual functional training systems and improving working conditions is associated with a lower risk of nursing home termination.

The nursing home termination rate in Japan is considerably lower than that in other countries, such as the United Kingdom (9) and United States (6, 8). The degree of market pressure in different countries can be attributed to differences in termination rates. In the United Kingdom, of the nursing homes that were closed down, one out of three closures were due to an oversupply of nursing homes or growth in alternative types of LTC provision (9). Likewise, in the United States, market competition was associated with nursing home termination (19, 20). However, the Herfindahl-Hirschman Index was not associated with nursing home termination in Japan, mainly due to the extreme shortage of nursing homes. Therefore, Japan has a potential market shock due to excessive demand even with a relatively low nursing home termination rate.

Nursing homes that offer physical function training are at lower risk of closing. In the past, nursing homes were living facilities where residents spent the last days of their lives. As an increasing number of nursing homes are offering functional training, they are gradually becoming a place where older adults can go to maintain their current living abilities to enable them to live well for a longer time. With this increasingly widespread awareness, older adults prefer nursing homes that offer this service, and nursing homes gain revenue consequently. To facilitate functional training in nursing homes, the Japanese government established an enhanced version of the functional training system with increased reimbursements in 2021 (19). We speculate that this financial incentive will encourage more nursing homes to initiate this service, although we were unable to quantify this in this study.

Nursing homes committed to improving working conditions (i.e., wage increases based on work experience and qualifications) were found to be less likely to terminate. Indeed, having clear expectations regarding wage progression in nursing homes was linked with job satisfaction (20). Using wage progression as a driver of motivation, more care staff may strive to obtain additional experience and qualifications. Further, a previous study reported a positive association between the improved working conditions for nurses and residents’ functional status (10). Thus, improved working conditions might enhance the quality of care and lead to the continual operation of nursing homes (13). Given the shortage of care workers and care work’s high job turnover rate in comparison to other industries, recruiting and retaining care workers is a major challenge for nursing homes. A previous study reported that the sharp decline in the number of caregiving jobseekers is due to the low wages in the care industry as compared to other service industries (21). Therefore, it is crucial to improve care workers’ compensation and secure the necessary number of caregivers to keep nursing homes operational.

Physical function training and staff retention could prevent nursing home termination; however, the requirements and implementation of the business operating systems must be considered in conjunction with the structural challenges of Japan’s LTC insurance system. Some policies have attempted to alleviate labor shortage and financial burden due to the rapid increase in the number of seniors older than 75 years; we did not include these changes in the discussion.

Our study has several limitations. First, the effects of the COVID-19 pandemic were not considered, even though part of the study was conducted during the relevant period. The number of nursing home terminations was 25, 33, and 27 in 2018, 2019, and 2020, respectively. The annual trend did not show the influence of the COVID-19 pandemic likely because only data for 2020, the beginning of the COVID-19 pandemic, was considered. A more extended study period which incorporates the COVID-19 pandemic may be appropriate to investigate the impact of the pandemic. In addition, the COVID-19 pandemic could have affected the business operating systems; thus, a panel data analysis defining business operating systems as time-variant variables would be desirable. Unfortunately, it was not possible to conduct panel data analysis in our study due to the small number of nursing homes that were terminated in each year. Second, residents’ medical conditions are a potential predictor of nursing home termination because they are directly related to difficulties in ensuring the appropriate number of medical staff and related management. This was not accounted for in the present study. Third, we were not able to consider staffing mix, such as registered nurse staffing. Fourth, our study did not capture the reasons for nursing home termination because of a lack of information. Fifth, logistic regression typically requires a large sample size; however, we only analyzed 83 terminated nursing homes, which is quite a small sample size. Further studies are needed to verify the consistency of our results with those of a larger sample. Sixth, the cross-sectional approach of the dependent variables limited our ability to make causal inferences from our findings. For example, nursing homes at risk of termination may not provide physical function training programs. Finally, a business operating system that results in a loss should not be allowed to continue to be used. We based our study on the assumption that implementing government-determined business operating systems would be profitable and affect ongoing operations. Future studies need to consider the net benefits of implementing business operating systems.

Despite these limitations, the strength of our study is that it provides evidence that business operating systems have an impact on nursing home termination. This study benefited from the Japanese universal LTC system, making nationwide examinations possible. The national LTC claims data included resident case-mix in each nursing home that was not controlled for in other studies. For internal factors, previous studies only considered total performance or deficiencies that were not comprehensive and which were difficult to develop a concrete plan around. In response, this study proposed specific measures for nursing home operations.

### Conclusion and Implications

Our study examined the impact of business operating systems on nursing home termination, after controlling for organizational, internal, and external factors. Implementing physical function training and improving care nurses’ working conditions were significantly associated with a lower risk of nursing home termination. With the continuing termination of nursing homes, additional reimbursements for specific business operating systems is a good option for policymakers aiming to build a sustainable nursing home environment.
